# Integration of Metabolic and Quorum Sensing Signals Governing the Decision to Cooperate in a Bacterial Social Trait

**DOI:** 10.1371/journal.pcbi.1004279

**Published:** 2015-06-23

**Authors:** Kerry E. Boyle, Hilary Monaco, Dave van Ditmarsch, Maxime Deforet, Joao B. Xavier

**Affiliations:** 1 Program in Immunology and Microbial Pathogenesis, Weill Cornell Graduate School of Medical Sciences, New York, New York, United States of America; 2 Program in Computational Biology, Memorial Sloan Kettering Cancer Center, New York, New York, United States of America; 3 Tri-Institutional Training Program in Computational Biology and Medicine, New York, New York, United States of America; University of Wisconsin-Madison, UNITED STATES

## Abstract

Many unicellular organisms live in multicellular communities that rely on cooperation between cells. However, cooperative traits are vulnerable to exploitation by non-cooperators (cheaters). We expand our understanding of the molecular mechanisms that allow multicellular systems to remain robust in the face of cheating by dissecting the dynamic regulation of cooperative rhamnolipids required for swarming in *Pseudomonas aeruginosa*. We combine mathematical modeling and experiments to quantitatively characterize the integration of metabolic and population density signals (quorum sensing) governing expression of the rhamnolipid synthesis operon *rhlAB*. The combined computational/experimental analysis reveals that when nutrients are abundant, *rhlAB* promoter activity increases gradually in a density dependent way. When growth slows down due to nutrient limitation, *rhlAB* promoter activity can stop abruptly, decrease gradually or even increase depending on whether the growth-limiting nutrient is the carbon source, nitrogen source or iron. Starvation by specific nutrients drives growth on intracellular nutrient pools as well as the qualitative *rhlAB* promoter response, which itself is modulated by quorum sensing. Our quantitative analysis suggests a supply-driven activation that integrates metabolic prudence with quorum sensing in a non-digital manner and allows *P*. *aeruginosa* cells to invest in cooperation only when the population size is large enough (quorum sensing) and individual cells have enough metabolic resources to do so (metabolic prudence). Thus, the quantitative description of *rhlAB* regulatory dynamics brings a greater understating to the regulation required to make swarming cooperation stable.

## Author Summary

Although bacteria are not multicellular organisms, they commonly live in large communities and engage in many cooperative behaviors. Cooperation can allow bacteria to access additional nutrients, but it requires the secretion of products that will be shared by the community. How bacteria make the molecular decision to cooperate within a community is still not completely understood. The bacterium *Pseudomonas aeruginosa* regulates the secretion of one of these shared products, rhamnolipids, using information about population density and nutrient availability in its environment. Expression of the operon *rhlAB* is required for the bacteria to produce rhamnolipids. We use a combined computational and experimental approach to investigate how *P*. *aeruginosa* continually combines current information of population density and nutrient availability to determine if it should express *rhlAB*. We find that when conditions are nutrient rich, *P*. *aeruginosa* uses population density to modulate the amount *rhlAB* expression, however when the bacteria are starved for nutrients the starvation condition largely determines how the bacteria will express *rhlAB*. Because the bacteria continually adjust expression based on the current conditions, the molecular decision to produce rhamnolipids can be adjusted if either population density or nutrient conditions change. Our combined computational and experimental approach sheds new light on the rich regulatory dynamics that govern a cellular decision to cooperate.

## Introduction

Cells can cooperate as multicellular populations and impact their environment in ways that would not be possible for an individual cell. This strength in numbers is observed in many natural populations of unicellular microbes and can be leveraged in engineered systems for synthetic biology [1]. While cooperation helps a population as a whole, natural selection acts at the level of individual cells, which makes cooperation susceptible to cheating [2]. The potential exploitation by non-cooperator cells (cheaters) that benefit from cooperation without participating in it threatens the robustness of multicellular systems both natural and synthetic [3–7]. However, the potential for cheating can drive the evolution of molecular mechanisms capable of effectively regulating cooperative traits [8]. Investigating the natural mechanisms that prevent cheating can reveal design principles underlying the robustness of multicellular systems.

In bacteria, the expression of cooperative genes is often regulated by density-dependent signaling systems, called quorum sensing, that detect the transition from a unicellular to a multicellular state [9–11]. Quorum sensing works by the secretion of small signaling molecules, called autoinducers, which accumulate in the extracellular space in a density-dependent manner. Cells sense the extracellular concentration of these molecules and use it as a proxy for population density. Quorum sensing is used to regulate the expression of cooperative multicellular traits such as bioluminescence [9], biofilm formation [12] and virulence factors [13–16]. The ubiquity of quorum sensing across bacterial species suggests a range of applications for this circuitry as a design principle [1,17]. Although quorum sensing can provide a robust benefit in changing conditions [18], it is vulnerable to cheating. Cheater cells can take advantage of quorum sensing autoinducers or public goods that are regulated by quorum sensing [3,19,20].

Cooperative genes regulated by quorum sensing can also be sensitive to nutrient conditions, suggesting that metabolic information is integrated into the decision to cooperate [21–28]. Integrating metabolic information with quorum sensing offers a possible mechanism to prevent cheating, as cells can only cooperate when they have the appropriate nutritional resources to do so, reducing the cost of cooperation to the individual cell. The opportunistic pathogen *Pseudomonas aeruginosa* secretes massive amounts of rhamnolipid biosurfactants in order to move collectively over surfaces, a phenomenon known as swarming [29–33]. Swarming provides a benefit at the population level, enabling cells in a colony to disperse over wide areas and grow to large numbers. However, rhamnolipid production can be a significant cost to the individual cell since it requires an investment of carbon that might otherwise be used for cell growth and division. Non-producing cells can exploit secreted rhamnolipids, which makes the trait vulnerable to cheating. Appropriate regulation of rhamnolipid synthesis is therefore crucial to prevent cheating and make cooperation stable.

The rhamnolipid synthesis operon *rhlAB* is regulated by a quorum-sensing cascade composed of *lasI/*lasR followed by *rhlI/rhlR* [34,35]. Although quorum-sensing regulation is necessary for *rhlAB* expression, it is not sufficient. Expression only occurs when the bacteria have carbon in excess of that needed for growth [23,36]. The use of metabolic signals to trigger expression of cooperative genes, in this case excess carbon triggering expression of *rhAB*, is termed metabolic prudence. This native regulation of quorum sensing and metabolic prudence prevents exploitation by cheaters and stabilizes cooperation [36,37].

The production of both mono- and di-rhamnolipids requires the function of three enzymes RhlA, RhlB and RhlC. RhlA converts B-hydroxyacl-ACP, an intermediate from fatty acid biosynthesis, into B-hydroxyalkanoyl-B-hydeoxyalkanoyl (HAA) [31]. RhlB and RhlC are required for the addition of rhamnose groups to produce mono- and di-rhamnolipids, respectively. RhlA is the rate-limiting enzyme and is required for any rhamnolipid production by the cell [38]. Tracking the activity of the *rhlAB* promoter therefore serves as a reporter for when a cell has made the decision to commit carbon to rhamnolipid production.

Although synthetic constitutive expression of *rhlAB* can result in rhamnolipid production and enable swarming, this synthetic rhamnolipid regulation severely impacts *P*. *aeruginosa* fitness and makes cooperation cheatable [36,37]. To understand how the native circuitry allows *P*. *aeruginosa* to produce rhamnolipids without compromising fitness we construct a quantitative picture of *rhlAB* regulatory dynamics. We combine quantitative experiments with mathematical modeling to systematically probe *P*. *aeruginosa* growth behavior and *rhlAB* promoter activity as a population transitions between different nutrient levels and population densities. We find that a classical Monod model cannot explain growth under starvation of nitrogen or iron and that internal pools of these nutrients sustain growth during starvation. Utilizing our understanding of growth in different nutrient limitations we quantitatively analyze *rhlAB* promoter activity and find that there are sufficient signals of excess carbon during exponential phase to trigger *rhlAB* expression in a density-dependent manner. We also find that although the limiting nutrient governs the qualitative behavior of promoter activity during starvation, starvation-induced activity is also scaled by population density. Together these results suggest a supply-driven activation that continually integrates metabolic prudence with quorum sensing in a non-digital manner. These results support the view that *P*. *aeruginosa* cells express rhamnolipids prudently to reduce fitness costs and prevent cheating and add details about the nuances of this regulation under different conditions.

## Results

### Expression of *rhlAB* Depends on Growth Phase

**Fig 1:**
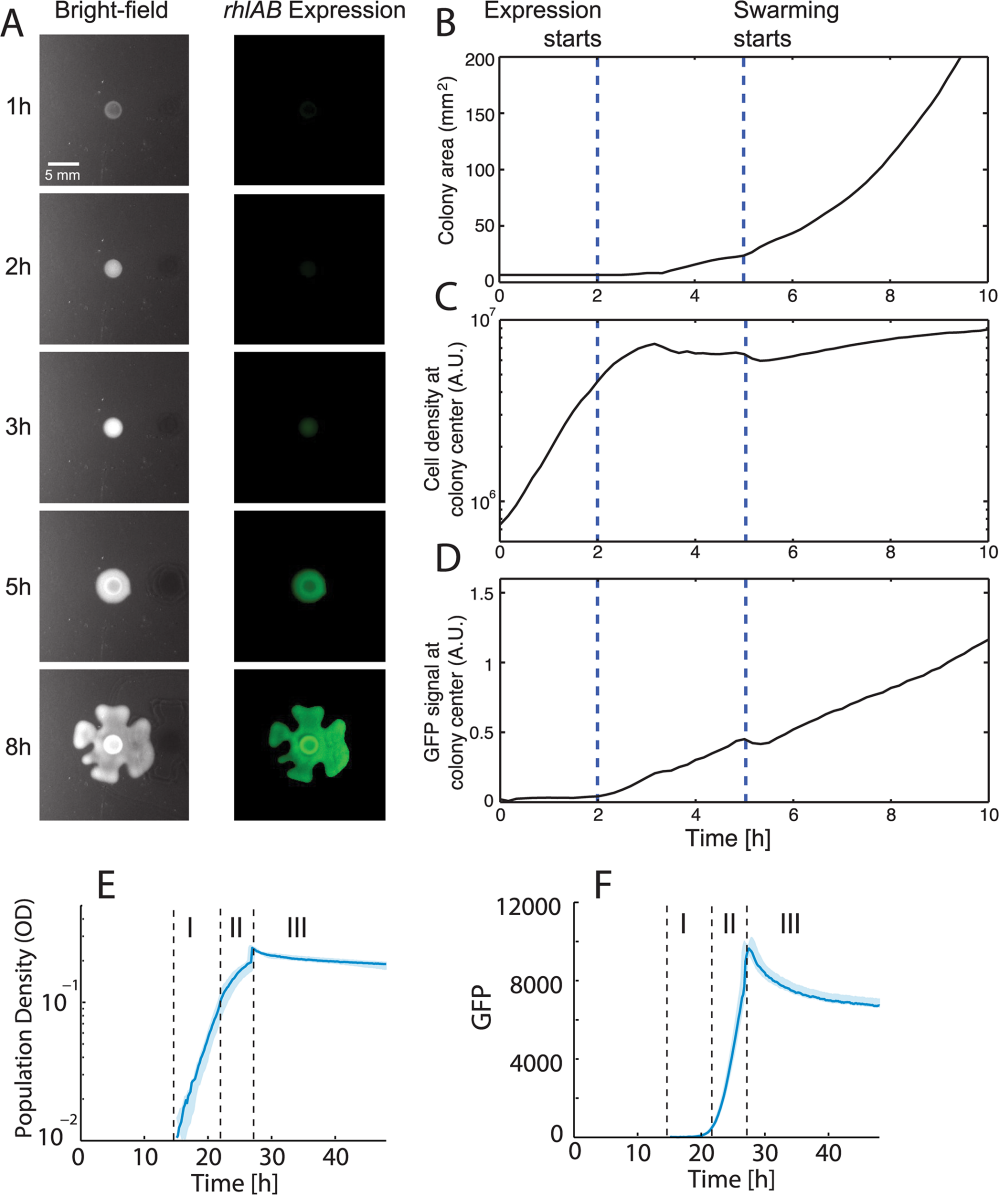
Expression of *rhlAB* coincides with a slowdown in growth. A. Time-lapse imaging of swarming and GFP fluorescence driven by the P_*rhlAB*_ promoter. The colony grows until it reaches a critical size at ~5h and subsequently begins tendril formation. Before tendril formation, *rhlAB* expression is observed. B-D The time points where *rhlAB* expression starts and swarming motility starts are indicated by dashed vertical lines. B. The increase in total area of the swarming colony shows that swarming starts at ~5h. C. Cell density at the center of the colony increases exponentially until t = ~2h then growth rate slows down D. The *rhlAB* expression (GFP signal) at the center of the colony was normalized by cell density. *rhlAB* expression revealed that expression of biosurfactant synthesis genes starts ~3h before the onset of swarming. E. Growth curve of a *P*. *aeruginosa* population in synthetic liquid media (see Synthetic Growth Media) where all three growth phases occur. Phases I, II, and III are indicated with dashed lines. F. P_*rhlAB*_–*gfp* expression of the population shown in E. over time. The majority of GFP production occurs during phase II, when the population growth rate has slowed. GFP measurements shown are corrected for autofluorescence (see Correction of Autofluoresence in the *gfp* Signal).

We analyzed the timing of *rhlAB* promoter activity directly in swarming colonies using fluorescent imaging and time-lapse video using a P_*rhlAB*_ *-gfp* reporter strain (Fig 1A) [39]. A colony of *P*. *aeruginosa* inoculated on a swarming plate first grows without moving until it reaches a certain critical size at ~5 h (Fig 1B). During the 2–5 h period expression at the colony center coincides with a decrease in growth rate that could be due to local nutrient depletion (Fig 1C and 1D). GFP levels continue to increase until ~5 h when we can observe a translucent ring of secreted rhamnolipids around the colony by eye. This is followed by the appearance of motile swarming tendrils shooting out from the colony. The secreted rhamnolipids lubricate the agar surface and allow the colony to slide over it.

In order to probe *rhlAB* expression more systematically, we turned to a batch culture system using shaken liquid media in a microtiter-based assay. This way we can simultaneously assess population density and gene expression using OD (optical density) and the P_*rhlAB*_ *-gfp* reporter respectively. Bacterial growth curves are typically described by four phases: lag phase, exponential phase (sometimes called “log” phase), stationary phase, and death phase. The definition of stationary phase often includes qualitatively distinct sub-phases of slowed growth and no growth, which can make analyzing responses to nutrient starvation difficult. To facilitate our analysis, we separate time series into three phases after lag phase. Phase I begins when the population becomes detectable by absorbance at 600 nm (OD, for optical density; detectable at 0.01 OD in this study) and grows exponentially at its maximum rate, μ_max_. During this initial period, the cells have all nutrients required for biomass synthesis and thus achieve balanced growth. The start of phase II is defined by growth limitation, where an essential nutrient runs out and the population growth rate has slowed below μ_max_. Phase III is when population density stops increasing and may actually decay. Fig 1E shows a representative growth curve with all three growth phases. The P_*rhlAB*_ *-gfp* construct enables tracking of *rhlAB* expression throughout the different phases of growth with high time resolution (Fig 1F). We corrected for *P*. *aeruginosa* secreted products that fluoresce in the GFP detection wavelengths, thus generating a compensated GFP signal from the P_*rhlAB*_ promoter over time (S1 Fig and Correction of Autofluoresence in the *gfp* Signal). Using population density and population level measurements of GFP assumes that *rhlAB* expression is homogenous across the population. To test if this assumption is valid, we used microscopy to measure single-cell expression levels at different stages of growth (S2 Fig). The up-regulation of *rhlAB* was simultaneous across the population rather than bimodal. Therefore, we concluded that the population-level measurements could be used to probe expression dynamics.

### Growth Response to Nutrient Limitation

**Fig 2:**
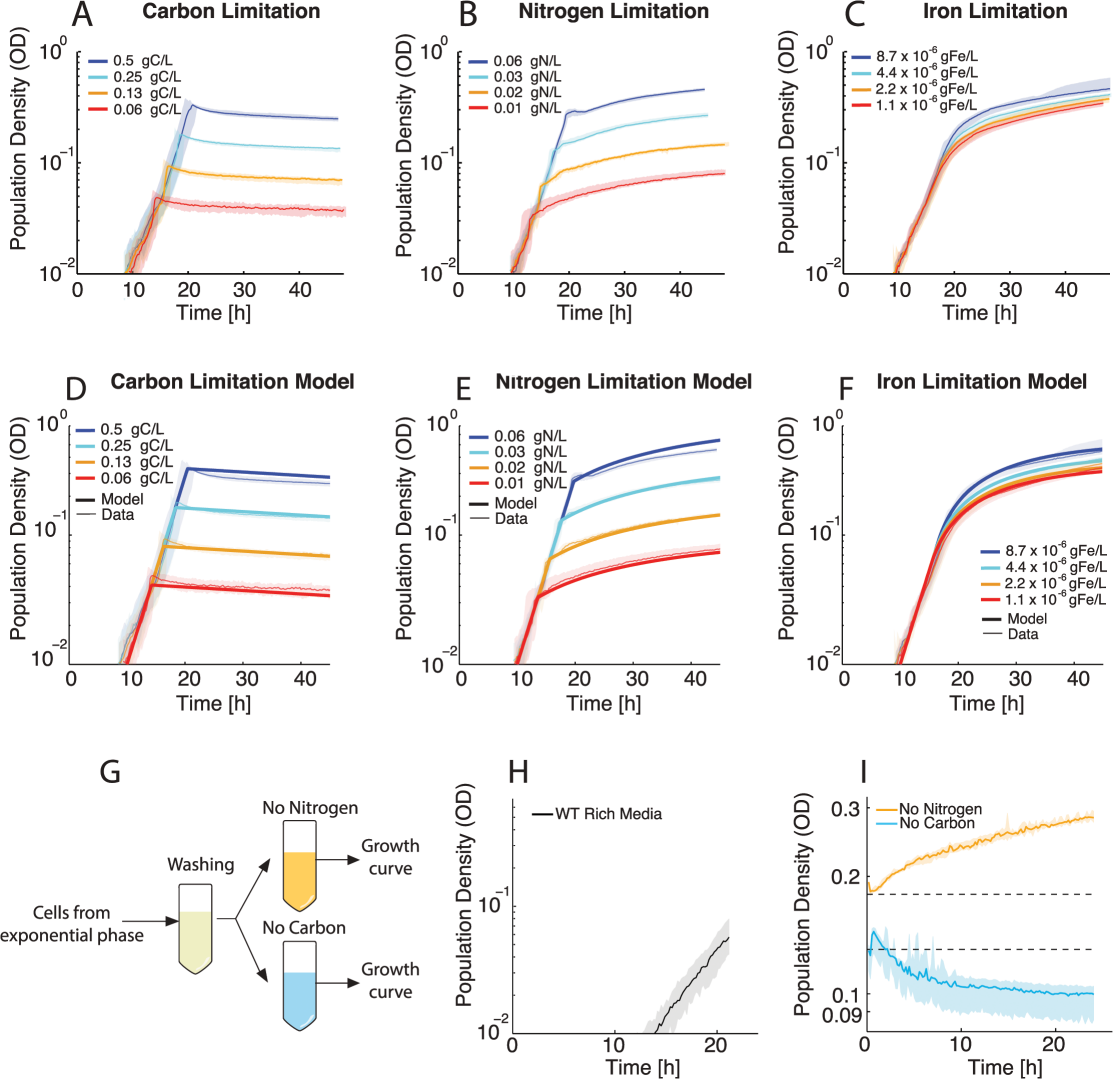
Bacterial growth behavior under different limiting conditions in batch culture. All growth curves are aligned to OD = 0.01 at 10 hours. See S2 Table for lag phase times. Shaded area represents full range; data lines represent the median. A. Growth of *P*. *aeruginosa* populations limited by carbon. The population transitions from phase I immediately to phase III when carbon is depleted with no observed period of slow growth. B. Growth behavior of populations limited by nitrogen. The populations abruptly transition from phase I to phase II when nitrogen is depleted. C. Growth behavior of populations grown in limiting concentrations of iron. The populations gradually transitions from phase I to phase II when iron is depleted from the media. For D-F the model is in thick lines and the data in thin lines. D. Mathematical model of growth in carbon-limited media. Growth halts when carbon is depleted from the media. E. Mathematical model of growth in nitrogen-limited media. Phase II growth is driven by an intracellular pool of nitrogen. F. Mathematical model of growth in iron-limited media. Phase II growth is driven by an intracellular pool of iron. G. Experimental scheme for testing growth behavior in the absence of extracellular carbon or nitrogen. H. Growth of the population that will be used in the depletion experiment. The population was grown to exponential phase in synthetic media rich in carbon, nitrogen and iron. I. Cells harvested from the population in H were washed and placed in one of the limiting media. The population without extracellular nitrogen is able to grow and increase in OD, while the population without extracellular carbon decays in OD. The data displayed is from a representative experiment from the biological replicates listed in S1 Table. The number of technical replicates is listed in S1 Table.

To better understand how a population responds to the entry into starvation we first analyze growth behavior in different limiting conditions. Our first growth limitation experiments set carbon as the growth-limiting nutrient in the media. We used varying concentrations of the carbon source (glycerol) and added a nitrogen source (ammonium sulfate) and iron (iron(II) sulfate) in excess. In these conditions, the population density and the length of phase I have a dose dependency with the initial amount of carbon, confirming that carbon is indeed the limiting nutrient (Fig 2A). Each growth curve follows the same phase I (exponential growth) with identical μ_max_ values (0.33 h^-1^). We observed that once the carbon in the media is fully consumed, cell growth stops abruptly and the population shifts sharply from phase I to phase III (decay) without going through a period of slowed growth (phase II). We carried out additional growth experiments, now in limiting concentrations of nitrogen. As before, population density and phase I time scale with initial nitrogen concentration, confirming that nitrogen is the limiting nutrient (Fig 2B). In nitrogen starvation the growth rate drops at the end of phase I but, unlike carbon starvation, population density continues to increase until the end of our observation period (approximately 45 hours). Throughout this period of slowed growth (phase II), the growth rate is continually decreasing.

Finally, we conducted experiments with iron as the growth-limiting nutrient. Increasing the amount of supplemented iron in the media increases both the population density and phase I, confirming that iron is the limiting nutrient (Fig 2C). Interestingly, the behavior under iron-limiting conditions was qualitatively distinct from both carbon and nitrogen limitation. In iron-limited growth there is still a transition from phase I to phase II, however this transition is gradual. The growth rate continually slows from μ_max_, unlike the abrupt transition to phase II seen in nitrogen limitation. In the iron limitation titration, there is an uneven spacing between the curves; doubling the supplemented iron did not result in a doubling of total population density. This can be explained by a constant yield for iron (Y_Fe_) and the presence of trace iron in the growth media. If trace iron is present then doubling the amount of supplemented iron will not double the total amount of iron in the system, but rather add to the trace amount already present. To test this explanation, we used the population density data from the iron titrations (Fig 2C), which fell on a line with slope Y_Fe_ (S3C Fig), to calculate the level of trace iron present in our non-iron supplemented media, Fe_0_ = 1.4 x 10^–5^ gFe/L. To additionally confirm the presence of trace iron in the media, we grew bacteria in media with no iron supplemented. As expected, the population was able to sustain a phase I (S4 Fig) in this medium and grew to the population density predicted by our trace iron calculation (S3C Fig). See S1 Table for information regarding biological and technical replicates.

### Mathematical Model Suggests Growth with Internal Nutrient Pools

In order to investigate how the nutrient levels experienced by cells influence their growth, we created a mathematical model of *P*. *aeruginosa* growth kinetics. Using the data from the experiments described above where we manipulated the media composition such that carbon, nitrogen, or iron become limiting, we were able to construct a kinetic model based on mass conservation, where biomass production is a function of these three nutrients. Central to this model was the calculation of the yields of biomass produced (in units of OD) per amount of carbon (Y_C_), nitrogen (Y_N_) and iron (Y_Fe_) consumed (S3 Fig). One benefit of this properly calibrated model is the calculation nutrient levels at any given time in the growth curve, which could not be directly measured with our assay.

Bacterial growth limitation due to depletion of an essential nutrient is commonly modeled using Monod kinetics, which requires two parameters: μ_max_, the maximum specific growth rate, and K_s_, the half-saturation constant [40,41]. In all of our nutrient limitations and titrations we observe the same μ_max_ in phase I independently of the initial nutrient concentrations. In order to satisfy this constraint, a Monod model would need to have a K_s_ value for each nutrient that is significantly below our lowest titration value (K_s_<<0.063 gC/L, K_s_<<0.0078 gN/L and K_S_<<1.4 x 10^–5^ gFe/L for carbon, nitrogen and iron respectively). In a Monod model, such a low K_s_ will give a very sharp transition from maximal growth to practically no growth. For carbon limitation we do observe this sharp transition behavior. This behavior is somewhat unexpected as the population might be predicted to slow in growth rate as carbon becomes increasingly scarce. This suggests that the half saturation constant value for carbon is indeed K_s_<<0.063 gC/L and in order to measure its actual value we would need to monitor population density at OD values below the detection limit of our growth curve assay (OD = 0.01). Therefore, we instead model carbon (C) consumption as a step function with a constant yield Y_C_ (S3A Fig and Eq (2)). This model recapitulates the growth behavior observed in carbon limitation media (Fig 2D).

Nitrogen and iron limitation growth curves, on the other hand, are inconsistent with a Monod model even with a K_s_ value below the lowest titration concentration. Although there is a sharp transition in nitrogen limitation from phase I to phase II, the sustained growth in phase II is incompatible with a Monod model. A Monod model with a low K_s_ also cannot explain the gradual slowdown in growth rate observed in iron limitation. There are a few possible explanations for the observed curvature in the nitrogen and iron limitations that we can exclude based on the data. Firstly, consistent phase II behavior across titrations excludes the possibility of a toxic product accumulating, as the populations with higher cell densities are not more severely affected. For nitrogen, the yield calculations predict no contaminating trace nitrogen amounts (S3B Fig). The possibility of a contaminating trace nitrogen that is used only in phase II is also eliminated as this would result in the populations with lower cell densities growing much more than the populations with higher cell densities for a given amount of trace nitrogen, and instead phase II behavior is consistent across the titrations.

The nutrient source for phase II growth in nitrogen and iron limitation must scale with population density and is independent of the starting nutrient concentration. A model that fits these criteria is one where the transition from phases I to II represents a switch in cellular metabolism from growth on extracellular nitrogen or iron (upon complete depletion of the limiting nutrient from the media) to intracellular nitrogen or iron [42]. Such growth behavior has been observed before in the yeast *Saccharomyces cerevisiae*. *S*. *cerevisiae* could grow in the absence of extracellular nitrogen by using nitrogen-rich intracellular biopolymers, presumably protein, and decreasing the nitrogen-to-carbon ratio of its biomass composition [43].

To determine if a model of *P*. *aeruginosa* utilizing internal nutrient pools for growth, like *S*. *cerevisiae*, could explain the observed behavior, we created a mathematical model to account for an internal nitrogen pool, internal iron pool, and trace iron. In this model, the cells consume extracellular nutrients to produce biomass and maintain homeostatic levels of intracellular nutrient pools while growing exponentially (N_i,_ Fe_i_) (Eq (3) and (5)). When the nitrogen or iron in the media is fully depleted, the cells switch to growth on the intracellular pool of the depleted nutrient, which then gradually decreases over time as the cells grow (Eq (4) and (6)). Because we cannot directly measure the size of the internal pool of nitrogen or iron, we normalize it by the size of the pool during balanced growth; both N_i,_ and Fe_i_ are dimensionless. Each cell enters phase II with N_i_ or Fe_i_ = 1 and this internal pool is then depleted for cell growth and diluted through cell division. The current growth rate of the population, μ(t), is dependent on the fraction of the internal pool in each cell. As the internal pool is depleted, growth slows from μ_max_ (Eq (7)).

The kinetics of biomass (X) (Eq (1)), growth and nutrient consumption of our different limiting nutrients are therefore given by:

$$\frac{dX}{dt}=\left\{ \begin{array}{l} \mu \left(t\right)X\;if\;C>0\\ \left(\mu \left(t\right)-k_{d}\right)X\;if\;C=0 \end{array}\right.$$ (1)

$$\frac{dC}{dt}=-\frac{1}{Y_{C}}\mu \left(t\right)X\;if\;C>0$$ (2)

$$\frac{dN}{dt}=\left\{ \begin{array}{l} -\frac{1}{Y_{N}}\mu \left(t\right)X\;if\;N>0\\ \;0\;if\;N=0 \end{array}\right.$$ (3)

$$\frac{dN_{i}}{dt}=\left\{ \begin{array}{l} -\left(\frac{1}{Y_{Ni}}+N_{i}\right)\mu \left(t\right)\;if\;N=0\;and\;N_{i}>0\\ \;0\;if\;N>0 \end{array}\right.$$ (4)

$$\frac{dFe}{dt}=\left\{ \begin{array}{l} -\frac{1}{Y_{Fe}}\mu \left(t\right)X\;if\;Fe>0\\ \;0\;if\;Fe=0 \end{array}\right.$$ (5)

$$\frac{dFe_{i}}{dt}=\left\{ \begin{array}{l} -\left(\frac{1}{Y_{Fei}}+Fe_{i}\right)\mu \left(t\right)\;if\;Fe=0\;and\;Fe_{i}>0\\ \;0\;if\;Fe>0 \end{array}\right.$$ (6)

where μ(t) is the current specific growth rate. Nitrogen and iron use the same internal nutrient pool model.

We find that in addition to accounting for trace iron in the media and internal nutrient pools of nitrogen and iron, we also must postulate that the maximum growth rate while using internal nitrogen is lower than μ_max_, here termed μ_max_´. This postulation is required to explain the sharp transition from phase I to phase II observed in nitrogen limitation (Fig 2B). As the transition in iron limitation is more gradual, this postulation is not required for growth on internal iron and thus we do not assume a switch from μ_max_ to an alternative value (μ_max_´) in iron limitation. Using this model, we are able to capture the dynamics observed in the experimental data (Fig 2D–2F). The equation set that determines μ(t) for the different nutrient conditions is given below

μ(t)=μmaxforC,NandFe>0μ(t)=0forC=0μ(t)=μmax×FeiforFe=0μ(t)=μmax'×NiforN=0 (7)

### Nutrient Depletion Experiment Supports Model with Internal Nutrient Pools

To test our internal nutrient model experimentally, we designed a nutrient depletion experiment (Fig 2G). The experiment was only performed for carbon and nitrogen as trace iron prevents the depletion experiment for iron. Cells were grown to exponential phase in rich synthetic media (Fig 2H) and then harvested while still in balanced growth, washed, and separately inoculated in media lacking either carbon or nitrogen. As predicted by our model, the cells in media lacking nitrogen were able to grow and increase in population density (Fig 2I) whereas in the absence of carbon, the population transitioned immediately to phase III and population density started to decrease (Fig 2I), supporting our model that total carbon depletion causes transition to phase III. These results are consistent with the model for phase II of nitrogen limitation and phase III of carbon limitation. We were unable to perform this experiment for iron limitation in the same manner due to the fact that medium without supplemented iron contains trace amounts of this nutrient (S3C Fig). Nonetheless, the model with an internal iron pool is still able to describe the observed growth behavior in iron limitation (Fig 2F).

In summary, we developed a model of *P*. *aeruginosa* kinetics that successfully captures the observed growth dynamics. In total, the model has eight free parameters, which we were able to parameterize using growth curve experiments to derive the nutrient yields, Y_C_, Y_N_, and Y_Fe_, as well as kinetic parameters (S5 Fig and S3 Table). We find that for carbon limitation, a Monod model (where growth slows with decreasing concentration of the limiting nutrient) with a very low K_s_ can explain the data, leading to a very sharp transition from exponential growth to no growth with virtually no slow down due to decreasing availability of carbon in the media (Fig 2D). The growth model also reveals that growth behavior in both nitrogen and iron limitation, under these conditions, is incompatible with an explanation using a Monod model. However, the behavior observed in these limiting conditions can be explained using a model of intracellular nutrient pools (Fig 2E and 2F), which is experimentally supported for nitrogen limitation (Fig 2I). A model using these intracellular nutrient pools is required to accurately capture bacterial growth dynamics and recapitulate the observed growth rate. This phenomenological model can be used to constrain the mechanisms of *rhlAB* expression responses we observe in nutrient limitations.

### *rhlAB* Promoter Response to Nutrient Limitation and Population Density

**Fig 3:**
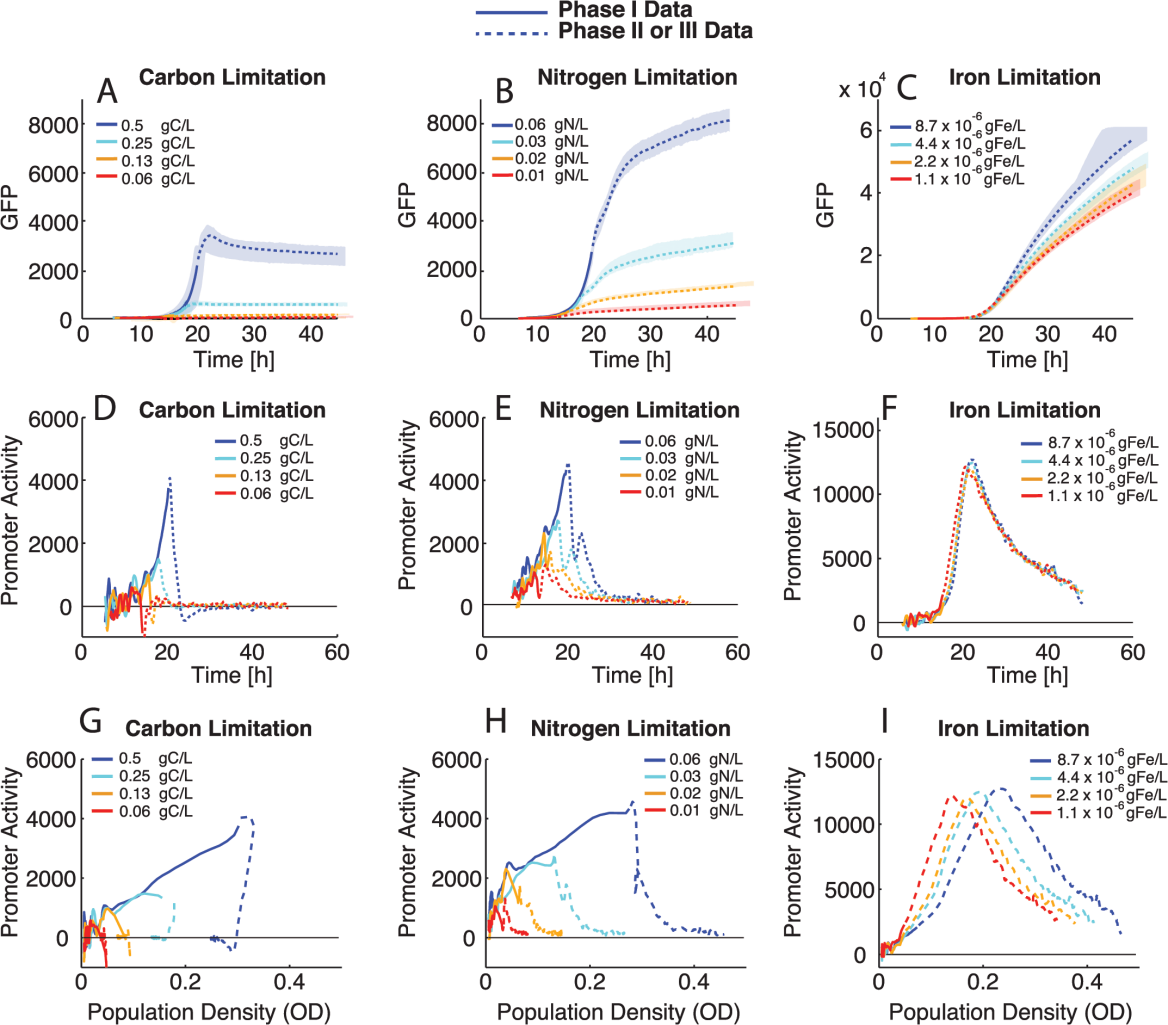
*rhlAB* promoter activity during different phases of growth in nutrient limitation batch culture. GFP and promoter activity data are from the same growth curves shown in Fig 2. Phase I GFP data and promoter activity are shown in solid lines while data corresponding to phase II or III are shown in dashed lines. Phases were determined by the mathematical model for bacterial growth discussed in the text. A. Population *rhlAB* (GFP) expression in carbon limitation media. Expression only occurs during phase I. B. Population *rhlAB* (GFP) expression in nitrogen limitation media. Expression occurs during both phase I and phase II. C. Population *rhlAB* (GFP) expression in iron limitation media. Expression occurs during phase I, but the majority of GFP production occurs during phase II. Iron limitation hits the saturation for GFP in our conditions ~ 6.0923 x 10^4^ Arbitrary fluorescence units. D-F P_*rhlAB*_ activity (see Promoter Activity Calculation) from growth curves in different limitation media D. In carbon limitation growth curves *rhlAB* promoter activity occurs during phase I and drops to zero during phase III for all titrations. E. In nitrogen limitation growth curves promoter activity occurs during phase I and is sustained during phase II for all titrations after an initial decrease at the end of phase I. F. In iron limitation growth curves promoter activity occurs during phase I and increases at the onset of phase II for all titrations. G-I *rhlAB* promoter activity from limitation growth curves plotted against population density (OD). G. Promoter activity from carbon limitation growth curves increases with density during phase I. H. Promoter activity from nitrogen limitation increases with population density during phase I and shows consistent qualitative behavior in phase II at different population densities. I. Promoter activity in iron limitation growth curves also increases with population density during phase I and shows consistent qualitative behavior in phase II at different population densities. Median data is shown in lines with the full range of technical replicates indicated by shaded area.

Rhamnolipid production is a dynamic process and changes in *rhlAB* expression coincide with transitions between growth phases, which are difficult to capture experimentally. Our growth model provides us with an understanding of the conditions cells experience throughout growth and the growth rate response to changes in the nutrient environment. We can use this understanding to interpret the expression response of *rhlAB* under these conditions. Our measurements of GFP driven by the *rhlAB* promoter (Fig 3A–3C) were taken simultaneously with the population density measurements (Fig 2A–2C) in the different limiting nutrient conditions. Using GFP (Fig 3A–3C) and OD measurements (Fig 2A–2C), we calculated the promoter activity for *rhlAB* throughout the time series (Fig 3D–3F), with compensation for GFP dilution by cell division (see Promoter Activity Calculation). Note that promoter activity fluctuates more at the early time points because there is more noise at low OD measurements due to technical limitations of the equipment. We use our mathematical model of growth to systematically identify when the population exits phase I (exponential phase). In Fig 3, expression during phase I is shown in solid lines while expression that occurs after phase I (phase II or III) is shown in dashed lines. Our study and model of growth behavior in this media revealed that entry into phase II or III indicates starvation by the limiting nutrient, therefore promoter activity that occurs after phase I occurs during nutrient starvation.

Previous work suggests that *rhlAB* expression occurs exclusively when carbon is in excess [23,36,44]. However, we unexpectedly observe appreciable *rhlAB* expression and promoter activity in carbon-limited media (Fig 3A and 3D and 3G). Expression in carbon-limited media occurs during phase I and promoter activity drops to zero when the population enters phase III. (The negative promoter activity values computed in carbon limitation media experiments (Fig 3D) are artifacts caused by a rapid increase in OD consistently occurring immediately before carbon starvation, coinciding with a shut off of *rhlAB* expression and beginning decay in the GFP signal.)

**Fig 4:**
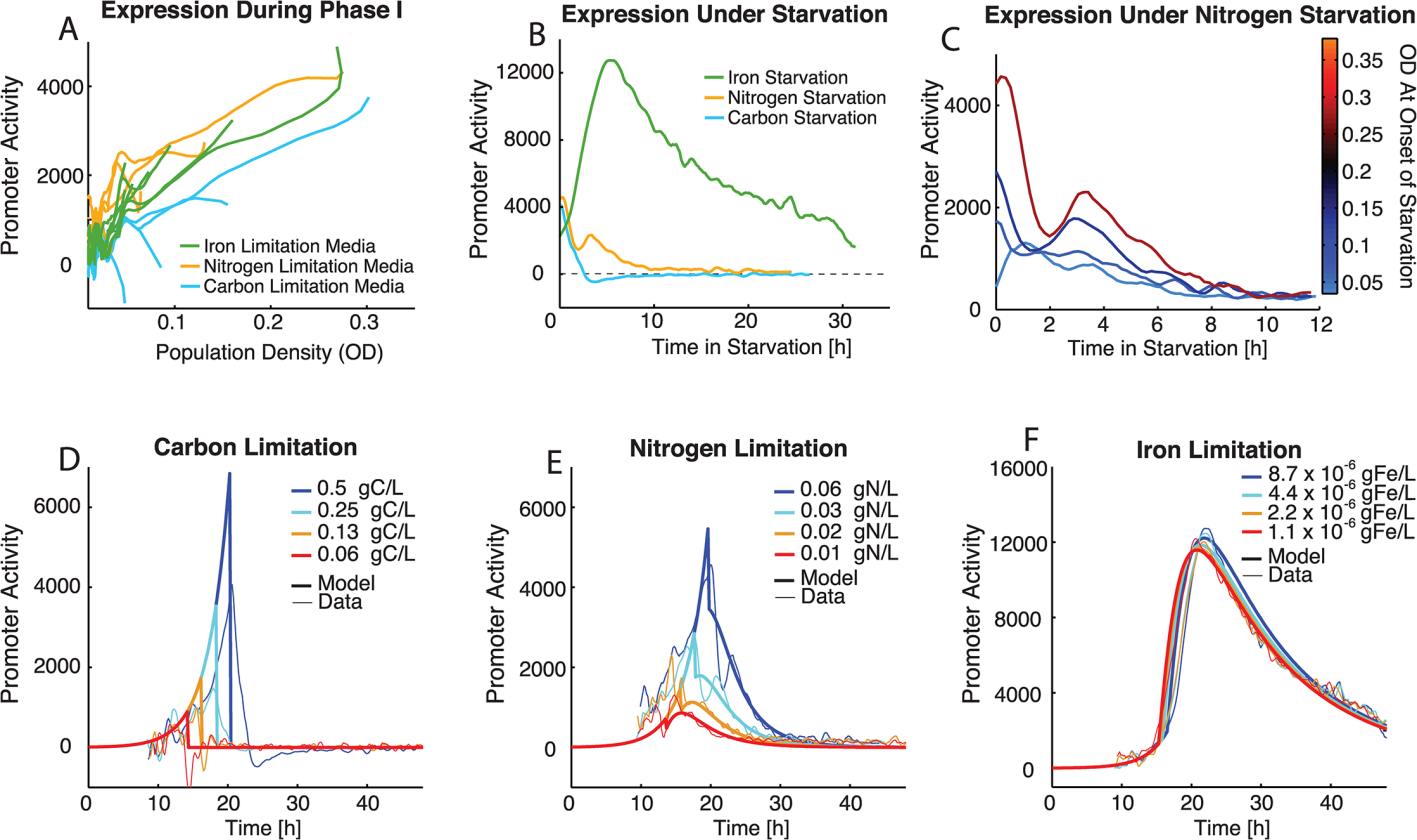
Quantitative analysis of *rhlAB* promoter dynamics and mathematical model of cooperation. A. Median of experimental data *rhlAB* promoter activity from phase I growth in different limitation media plotted against population density (OD). A similar slope is observed for all limitation media suggesting a consistent relationship between population density and *rhlAB* promoter activity. B. Median *rhlAB* promoter activity during growth under nutrient starvation over time. *rhlAB* promoter activity increases in iron starvation, is sustained in nitrogen starvation and is shutdown in carbon starvation. The mathematical model of growth systematically determined the start of starvation. Iron starvation initial condition 8.7 x 10^–6^ gFe/L, nitrogen starvation initial condition 0.6 gN/L, carbon starvation initial condition 0.5 gC/L, all shown in Fig 3D–3F. C. Median *rhlAB* promoter activity from phase II of nitrogen limited populations (Fig 3E). Populations with higher density at the onset of starvation have higher *rhlAB* promoter activity during nitrogen starvation. D-F Mathematical model of *rhlAB* promoter activity compared to experimental data. The model is shown in thick lines and median experimental data is shown in thin lines. A model integrating nutrient starvation and population density is able to capture the many aspects of *rhlAB* promoter activity during periods of balanced and limited growth. D. Carbon limitation media. E. Nitrogen limitation media. F. Iron limitation media.

It is interesting that promoter activity increases throughout phase I, as it could be expected that there would be a constant level of expression during balanced growth and promoter activity would equilibrate quickly. However, increasing promoter activity during phase I is observed in all three limitation conditions (Fig 3D–3F, solid lines). This phase I promoter activity could be due to the fact that even during exponential growth there is carbon available in excess of what is needed for biomass production, and this carbon can be dedicated to rhamnolipid synthesis. The promoter activity in phase I increases with population density in all conditions suggesting that this expression is density dependent (Fig 3G–3I). By overlaying phase I of all conditions and plotting against population density we confirm a consistent slope across all conditions, suggesting that the mechanism driving the density dependent expression is the same in all three limitation conditions (Fig 4A).

In nitrogen limited media there is not a complete shutdown of expression after phase I. Instead, population level GFP continues to increase in phase II (Fig 3B). The promoter activity drops, but is sustained, with a second peak of activity occurring before tapering off to near zero (Fig 3E). In contrast, *rhlAB* promoter activity during phase II of iron limitation rapidly increases without an initial drop when the population enters phase II. Promoter activity of *rhlAB* decreases over time, but never reaches zero in our iron limitation condition (Fig 3F). The level of promoter activity reached in iron limitation is much higher than that in nitrogen limitation when the highest titrations are compared directly (Fig 4B). We also observe that even in nitrogen and iron starvation when promoter activity is sustained or induced, activity eventually shuts down, most likely due to the prolonged starvation experienced by the cell.

Similar behavior is observed for all titrations within each limiting media across a wide range of population densities (Fig 3G–3I). Therefore the limiting nutrient and the duration of starvation appear to be the main drivers of qualitative promoter behavior: shut down of activity in carbon starvation, sustained activity in nitrogen starvation, and induction of activity in iron limitation (Fig 4B). Although the qualitative behavior is determined by the limiting nutrient, a closer examination of promoter activity during nitrogen starvation reveals that populations with a higher density have higher *rhlAB* promoter activity even during starvation (Fig 4C). This suggests that the cells continue to use density-dependent information to modulate the dynamics of *rhlAB* promoter activity in nitrogen limitation.

### Phenomenological Model of the Cellular Decision to Cooperate

To determine if our current understanding of the *rhlAB* promoter response was sufficient to explain the observed dynamics, we derived a mathematical expression of P_*rhlAB*_ *-gfp* promoter activity. The model contains three components. The first component implements the density-dependent up-regulation observed during balanced growth (Fig 4A). The second component implements the observation from here, and in previous work, that *rhlAB* is expressed under nutrient starvation when growth is limited, but not halted (Fig 4B, nitrogen and iron starvation) [23,36]. Since promoter activity under starvation is also a function of population density (Fig 4C), we use an additive model to describe the integration of population density and nutrient starvation induction. The third component implements a decrease in promoter activity that is observed under prolonged starvation by either nitrogen or iron (Fig 4B). Prolonged starvation shutdown is implemented using Hill kinetics.

Our mathematical model of bacterial growth predicts not only nutrient levels over time, but also the effect of these nutrient levels on growth rate. With an accurate prediction of μ(t), from the growth model, and an understanding of the nutrient environment the cells experience in phases II and III, we are able to use growth rate as an indicator of cell starvation. Therefore, expression under starvation is implemented by induction when μ(t) falls below μ_max_ (μ_max_’ for nitrogen starvation)_._ Promoter activity only occurs when carbon has not been depleted in the media. By using μ(t) as an indicator of starvation, rather than absolute nutrient values, our model remains flexible and can be adapted to multiple limitations of these nutrients or additional limiting nutrients. Two variables are required for converting the expression to GFP units and scaling the different components. The variable q_D_ is used to scale the density dependent activity component and is the same value for all nutrient conditions. The variable q_R_ is used to scale the starvation induced promoter activity. Consistent with our observations that expression under nutrient starvation depends on the limiting nutrient (Fig 4B), different values of q_R_ are required to achieve the observed levels of activity in nitrogen or iron limitation (q_RN_ and q_RFe_ respectively)

Activity during prolonged starvation shuts down progressively when μ(t) has decreased further and falls below a threshold fraction of μ_max_, define here as k_g_. We find again that the limiting nutrient has a great effect on promoter activity and to implement the appropriate shutdown in both nitrogen and iron limitation the values for k_g_ and h must be adjusted for each limitation (k_gN_, h_N_ and k_gFe_, h_Fe_ respectively). Although we must adjust for the different starvation conditions of nitrogen and iron, a parameterized three-component model of *rhlAB* expression (Eq (8)) is able to recapitulate the observed expression dynamics under our different nutrient limitations (Fig 4D–4F).

$$P_{rhlAB}=\left(q_{D}X+q_{R}\left(\frac{\mu_{\text{max}}\left('\right)}{\mu }-1\right)\right)\times \frac{1}{1+{\left(k_{g}\times \frac{\mu_{\text{max}}}{\mu }\right)}^{h}}$$ (8)

Although it is perhaps at first unsatisfying that the fitted parameters must be changed to account for behavior in both nitrogen and iron starvation, this ultimately supports that the internal state of the cell, which drives *rhlAB* promoter activity, is significantly different in these two conditions. By eye it is not clear if the growth rate and population size differences could be sufficient to explain the different behavior in the two starvation conditions. However, even by taking the observable differences into account we found no model or single parameter set that could sufficiently explain promoter activity in both starvation conditions simultaneously.

We find that this model is capable of capturing the observed dynamics and indicates that the metabolic signal for expression is potentially at different levels during nitrogen and iron starvation or that downstream regulation is different in these two limitations. The use of μ(t) from our growth model functions as an indicator of starvation and accurately predicts the response of the *rhlAB* promoter to nutrient starvation. Importantly, both density-dependent regulation and metabolic regulation are required in the model as non-digital regulatory components to recapitulate the observed expression dynamics in all nutrient conditions.

### Experimental Test of the Role of Quorum Sensing

**Fig 5:**
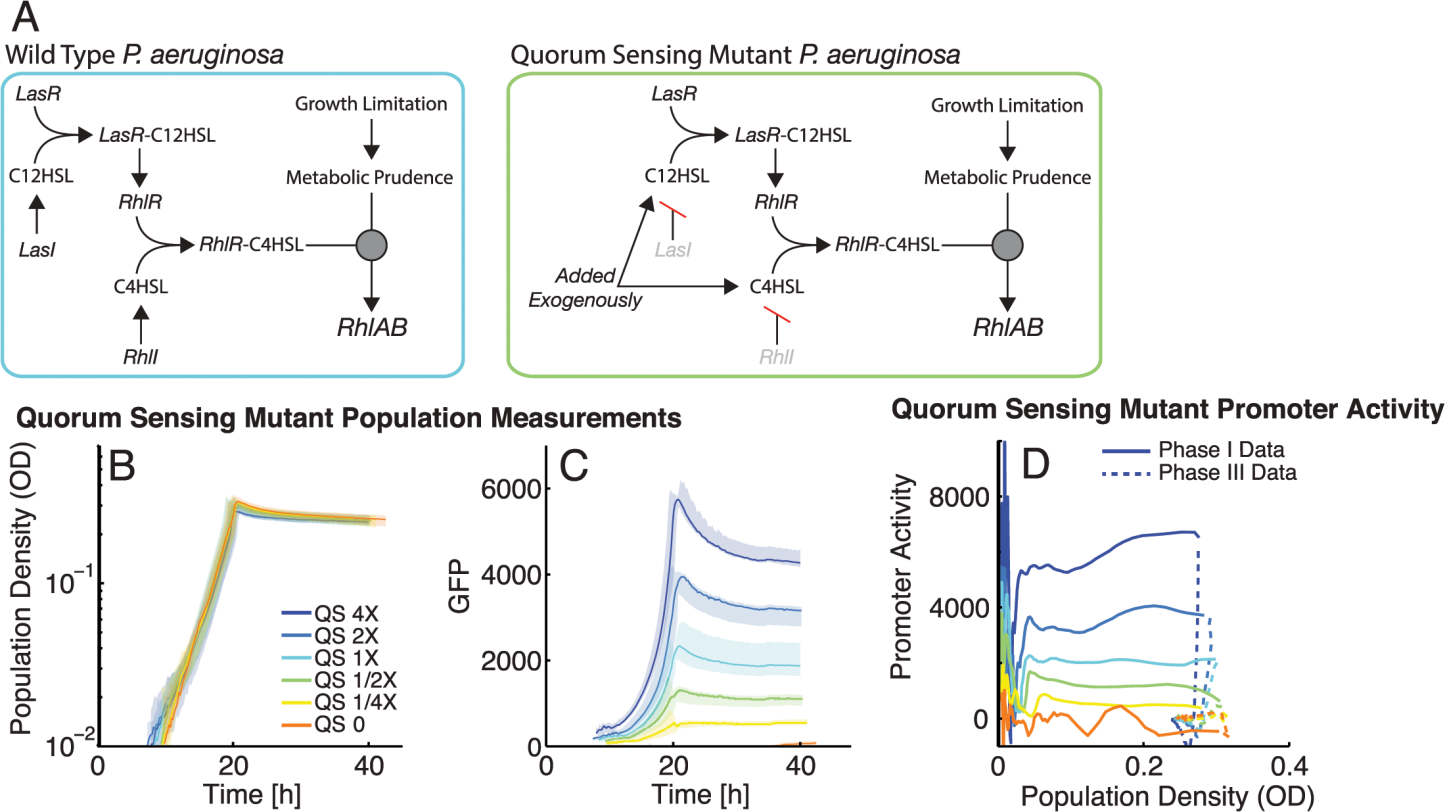
Density-dependent scaling of *rhlAB* expression is controlled by quorum sensing autoinducers. A. Quorum sensing regulatory cascade in *P*. *aeruginosa* WT and in the quorum-sensing mutant, which lacks the genes encoding LasI and RhlI. Median is shown in thick lines with full range indicated by shaded area. All growth curves are aligned to OD = 0.01 at 10 hours. See S2 Table for lag times. B. Growth of the ∆*lasI*∆*rhlI* bacterial populations in carbon limitation media with 0.5 gC/L and different concentrations of auto inducer 1 X = 1 μM C12HSL and 5 μM C4HSL. 1X is estimated to be physiological and has recovered the WT level of rhamnolipid production in previous work [19]. C. GFP expression for each population. Populations that received higher levels of autoinducer express higher levels of GFP. D. P_*rhlAB*_ activity in different concentrations of autoinducer plotted against population density. Activity holds constant during phase I and rapidly shuts off at the onset of phase III. Phase I is shown in solid lines and phase III in dashed lines.

Previous work has shown that quorum sensing is required for *rhlAB* expression, however our observation of a gradual increase in *rhlAB* promoter activity as population density increases (Fig 4A) suggests a more nuanced role for quorum sensing in *rhlAB* expression. To confirm that quorum sensing does mediate the density-dependent component of *rhlAB* expression and to explore the effect of perceived density on *rhlAB* promoter activity, we utilized a quorum sensing mutant that does not produce the *lasI/lasR* nor *rhlI/rhlR* system autoinducers C_12_HSL (*N*-(3-oxododecanoyl)-L-homoserine lactone) and C_4_HSL (*N*-butyryl-L-homoserine lactone), respectively and we manipulated the levels of autoinducers in the medium. This strain (PA14 ∆*lasI* ∆*rhlI attB*::P_*rhlAB*_ *-gfp*) has the same P_*rhlAB*_ *-gfp* reporter as our wild-type strain (Fig 5A).

We first performed an extensive test of *rhlAB* induction across a wide range of autoinducer concentrations by varying the concentrations of each autoinducer independently (S6 Fig). The data revealed that although one autoinducer can compensate for a lack of the other, this requires very high levels of that single autoinducer, which are likely not biologically relevant. We also observed that when both autoinducers are increased in concentration, but kept in the same proportion, there is a consistent increase in *rhlAB* expression. We proceeded with a fixed 1:5 ratio of C_12_HSL to C_4_HSL used previously [36]. To isolate the density-dependent component of expression, the ∆*lasI*∆*rhlI* strain was grown in carbon-limited media complemented with different concentrations of C12HSL and C4HSL kept at a 1:5 ratio (Fig 5B).

Higher total levels of *rhlAB* expression were observed with higher concentrations of quorum sensing signal (Fig 5C). Importantly, promoter activity of *rhlAB* became constitutive during phase I (exponential growth) confirming that quorum sensing modulates expression during that phase (Fig 5D). In contrast to promoter activity of the wild-type PA14 strain (WT) during exponential growth, phase I promoter activity in the mutant is decoupled from population density (Fig 5D, compared with Fig 4A). The constitutive level of promoter activity in the mutant scales with the concentration of the autoinducers in the medium, as predicted from the relationship between population density and *rhlAB* promoter activity we observed and modeled in the WT.

We hypothesize that this phase I promoter activity behavior is the result of quorum sensing signals inducing promoter activity when there is a constant level of carbon-rich metabolites present inside the cell during balanced growth in this medium. Because the level of carbon-rich metabolites is constant, promoter activity is modulated only by changes in population density, sensed by quorum sensing signals. Quorum sensing regulation of *rhlAB* is confirmed here to not be a digital switch, but instead produces a graded *rhlAB* expression response. Also, the shutdown of *rhlAB* promoter activity due to carbon depletion occurs even in the presence of high levels of quorum sensing autoinducers (Fig 5D) demonstrating that carbon starvation is capable of overriding the quorum-sensing regulated induction. Using this mutant strain we were also able to confirm that quorum sensing signals scale *rhlAB* promoter activity during starvation by nitrogen or iron (S7 Fig).

### Swarming Cooperation in Nutrient Limitation

**Fig 6:**
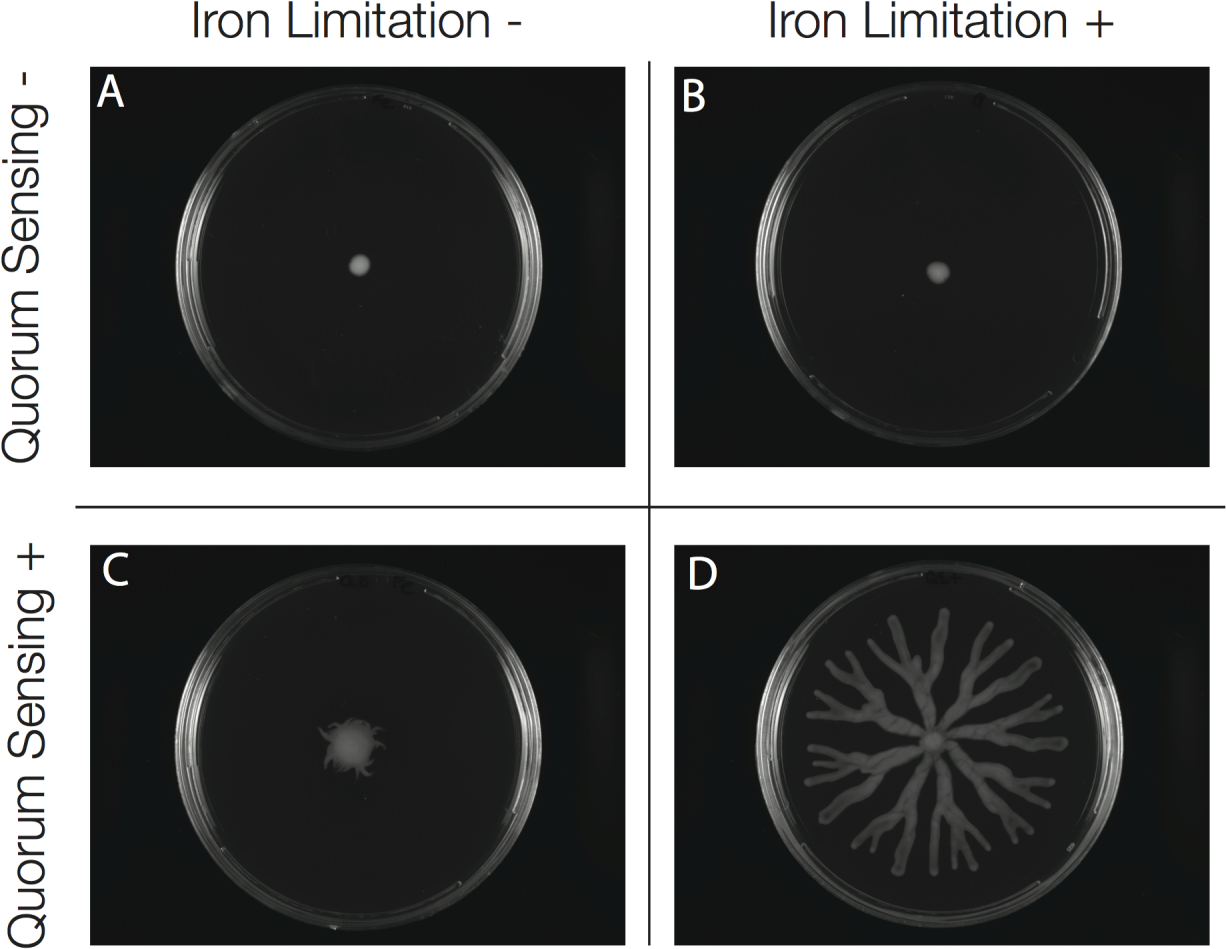
Quorum sensing signals and iron limitation are required for swarming colony formation. Quorum sensing signals and iron were supplemented in the agar swarming plates. Quorum sensing signals were supplemented at 1 μM C12HSL and 5 μM C4HSL. Iron was supplemented at 2.79*10^–4^ gFe/L by addition of iron(II) sulfate. All swarms were done using the ∆*lasI*∆*rhlI* strain A. Populations that do not receive quorum sensing signals and are not in iron limiting conditions fail to swarm. B. Populations that do not receive quorum sensing signals and have iron limiting conditions fail to swarm. C. Populations that receive quorum sensing signals, but are not in iron limiting conditions do not swarm far from the inoculation site and do not form branching tendrils. This demonstrates the key role of iron limitation in rhamnolipid production and swarming colony formation D. Populations that receive quorum sensing signals and have iron limiting conditions exhibit WT swarming colony morphology with branched tendrils that extend to the edges of the plate.

We were able to identify the differential responses of the *rhlAB* promoter to different nutrient limitations and population densities in our liquid culture system. To test the effects of the identified *rhlAB* promoter responses on swarming cooperation, we grew swarming colonies of the ∆*lasI*∆*rhlI* quorum sensing null strain in different media conditions. Unlike in liquid culture experiments, the media used in swarming assays has to be a complex media where casamino acids serve as the carbon and nitrogen source. In our liquid culture system, we found iron limitation to be a potent inducer of *rhlAB* activity. To test if the integration of quorum sensing signals and iron limitation is required for swarming colony formation we tested several conditions with and without iron limitation and with and without quorum sensing signals. Without quorum sensing signals, the colony is unable to swarm regardless of whether iron is limiting (Fig 6A and 6B). As predicted from our liquid culture experiments and mathematical models, if a population has quorum sensing signals but lacks iron limitation, the colony does not have normally branching and does not travel far from the inoculation site (Fig 6C). This confirms that the significant induction of *rhlAB* promoter activity we observe under iron limitation in our liquid culture system is also key for rhamnolipid production in swarming colonies. Only when a population is provided with both quorum-sensing signals and iron limitation does successful swarming occur (Fig 6D). Swarming behavior in these four conditions supports our liquid culture data; significant production of rhamnolipids requires both quorum sensing signals and iron limitation. The observation that iron starvation facilitates swarming cooperation is consistent with our experiments showing that iron starvation induces higher *rhlAB* promoter activity than nitrogen starvation or quorum sensing signals alone (Fig 3F) and with previous reports [45,46]. Given these data the induction of *rhlAB* expression in swarming colonies (Fig 1D) is likely induced by iron limitation. We also tested the effect of nitrogen limitation, iron limitation, and additional quorum sensing signals on the WT. We found again that iron limitation is necessary for successful swarming while nitrogen limitation and additional quorum sensing signals only moderately affect swarming colony morphology (S8 Fig).

## Discussion

Here we investigated the molecular circuitry underlying the regulation of genes required for a model multicellular trait, swarming in *P*. *aeruginosa*. We performed this analysis using a combination of quantitative growth curve experiments and mathematical models. Swarming requires cooperation between cells and the production and secretion of rhamnolipids [32]. Expression of the *rhlAB* operon results in expression of the rate-limiting enzyme for rhamnolipid synthesis and commits the cell to cooperation [31,38]. We carried out liquid batch culture growth experiments in shaken microtiter plates, which allows for a high-throughput investigation of gene expression during periods of changing nutrient conditions and cell densities [47]. Shaken liquid-culture neglects the spatial gradients of rhamnolipids, quorum sensing signals and nutrients that may occur in swarming colonies [48,49]. In turn, the conditions experienced by cells can be more precisely manipulated in liquid culture allowing us to build a quantitative picture of how metabolic prudence and quorum sensing are integrated into the cellular decision to cooperate.

Our data and mathematical model support previous metabolic prudence models where the expression of *rhlAB* is triggered by excess carbon. During balanced growth internal levels of carbon-rich metabolites are constant and *rhlAB* promoter activity increases proportionally to population density (quorum sensing signals). Since the population maintains the same growth rate in spite of increasing *rhlAB* promoter activity, the uptake rate of carbon would be predicted to also increase, to compensate for the increasing demand of rhamnolipid synthesis. This suggests that the rate of carbon uptake is not limiting the growth rate during balanced growth and that even in exponential growth cells can increase carbon uptake to allow for rhamnolipid synthesis.

When carbon is fully depleted, *rhlAB* expression stops abruptly, potentially to reduce the demand on intracellular carbon-rich metabolites when the lack of carbon has become growth limiting. When extracellular iron is depleted, growth slows and *rhlAB* expression increases. We predict that the decreased growth rate reduces the demand for carbon in biomass production, leading to excess carbon-rich metabolites, which in turn trigger *rhlAB* expression. Growth also slows during nitrogen starvation, which should also decrease the demand for carbon in biomass production. However, cells can actively decrease carbon uptake due to nitrogen starvation [50], which would balance the decreased demand for carbon in biomass production. In support of this balancing of supply and demand we observe that the expression of *rhlAB* during nitrogen starvation is sustained at a low level, but does not increase. Also in support of nitrogen starvation actively decreasing carbon uptake more significantly than iron starvation, our mathematical model reveals that maximum growth rate must undergo a dramatic decrease in nitrogen starvation, but not in iron starvation.

Quorum sensing regulates multicellular traits in natural and synthetic bacterial systems, but it is not sufficient for robustness against cheating [3,18–20]. Promoter activity of *rhlAB* during exponential growth and non-carbon nutrient starvation scales with population density, supporting that quorum sensing signals do not act as a checkpoint, but instead continually modulate promoter activity and the decision to cooperate through swarming. Our results demonstrate that these two regulatory mechanisms are continually integrated throughout the entire period of expression and may allow cells to adapt to fluctuations in nutrient conditions and population concentration. The ability to respond to changing population density after the decision to express *rhlAB* has been made could play a role in maintaining an individual’s fitness while cooperating in a mixed population [37]. Furthermore, metabolic prudence was recently demonstrated as a regulatory mechanism in multiple other secreted products of *P*. *aeruginosa* [23], suggesting that integration of population density and nutrient environment information may be a more widespread regulatory strategy.

**Fig 7:**
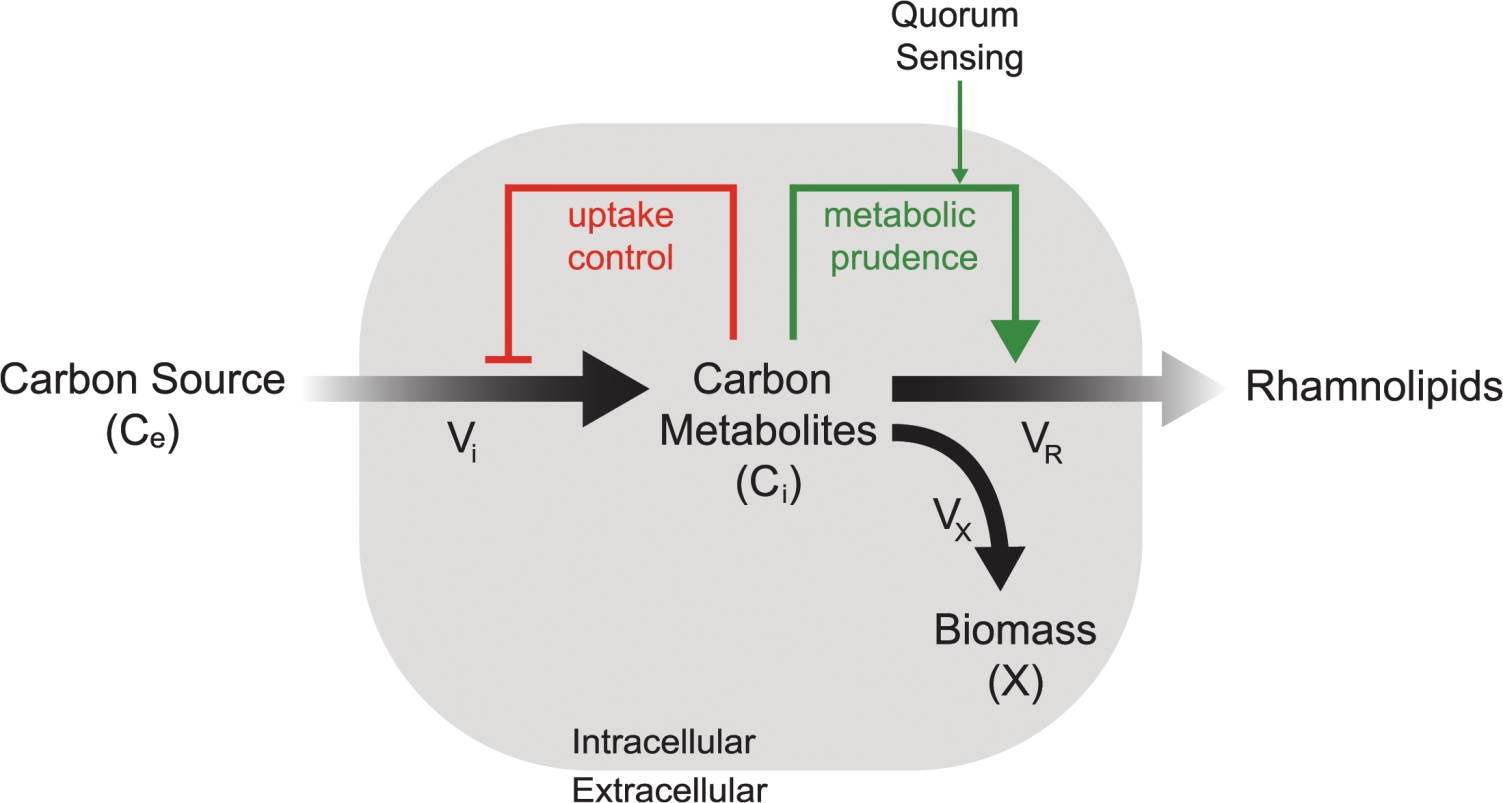
Conceptual model of cooperation by rhamnolipid secretion. Conceptual feed-forward supply-driven model of growth and *rhlAB* expression in *P*. *aeruginosa*. A slowdown in growth would lead to buildup of intracellular carbon metabolites. This buildup would trigger the expression of genes, in this case *rhlAB*, which would convert carbon metabolites into secreted rhamnolipids that can benefit the cell and the population. By only expressing *rhlAB* when there is a buildup of metabolites the cell never decreases the rate of biomass production, V_*x*_.

Taken together our data suggests that rhamnolipid synthesis is regulated by feed-forward supply-driven activation, similar to the coupling of end-product inhibition and supply-driven activation reported to regulate amino acid pools in *E*. *coli* [51]. To ensure maintenance of intracellular carbon rich metabolites, the uptake rate of carbon is in turn regulated using a feedback end-product inhibition (Fig 7). The data presented here supports that quorum sensing is continually integrated into the decision to express *rhlAB* and that the cell is capable of maintaining a constant rate of biomass production even while expressing *rhlAB* during exponential phase. The latter observation suggests that the cell can increase the uptake of carbon during balanced growth to compensate for the production of rhamnolipids.

Our analysis provides new insights into metabolic prudence, but many details of its molecular implementation remain to be discovered. For example, what is (are) the molecule(s) that indicates that intracellular carbon is in excess? Which proteins detect these metabolic signals and how do they interact with quorum sensing regulation at the molecular level? Answering these and other questions will improve our knowledge of rhamnolipid synthesis by *P*. *aeruginosa* with implications for medicine and industry. Rhamnolipids have the power to disperse infectious biofilms of *P*. *aeruginosa* and other bacteria, and could thus be used in medical applications [52,53]. They also have commercial value as biodegradable surfactants [54,55]. Beyond *P*. *aeruginosa*, metabolic prudence emerges as a design principle to stabilize cooperation in multicellular groups. Like other design principles of biology [56] metabolic prudence may be applied to synthetic biology where quorum sensing modules are already used to regulate population level traits [57,58].

## Materials and Methods

All chemicals were obtained from Fisher Scientific (Waltham, MA) unless otherwise specified.

### Single Cell Gene Expression

Cells of the P_*rhlAB*_ *-gfp* reporter were taken from a growth curve in a 96 well plate using the growth curve synchronization method described previously [59]. Cells were taken from the 96 well plate wells and mounted on agar pads of low melt agar (approximately 1.75% agar) with no nutrients. Cells were imaged for bright-field, GFP, and DsRed. A ratio of GFP to DsRed was used to determine expression level of *rhlAB* by GFP.

### Correction of Autofluoresence in the *gfp* Signal

To correct for autofluorescence in the GFP signal, we measured the fluorescence of the unlabeled PA14 strain, which lacks the P_*rhlAB*_-*gfp* reporter, in numerous experiments across multiple days and a range of conditions, and performed a linear regression on the logarithm of these data (S1A Fig). We found the OD data (OD_600_) to be a good predictor of the autofluorescence. The fit of the autofluorescence (AF) is then a function of the OD_600_ of the form:

$$AF=e^{\left(a+b\times \text{log}\left(OD_{600}\right)\right)}$$

We found a correlation coefficient r^2^ = 0.97 using all data points that were OD_600_>0.01. Using this function, we estimated the amount of autofluorescence in our reporter strains by using the OD_600_. We then subtracted the calculated autofluorescence value from the total GFP signal to obtain a corrected GFP (S1B Fig).

### Synthetic Growth Media

All synthetic media utilized glycerol as the sole carbon source, ammonium sulfate as the sole nitrogen source and Fe(II) sulfate (Acros Organics, Geel, Belgium) for iron supplementation. Base media contains 64 g/L of Na_2_HPO_4_.7H_2_O, 15 g/L of KH_2_PO_4_, 2.5 g/L of NaCl, 1 mM of MgCl_2_, 0.1 mM of CaCl_2_ and carbon, nitrogen and iron concentrations depending on the limiting nutrient. Carbon limitation media: 0.5 gN/L, 2.79 *10^–4^ gFe/L, carbon at listed concentration. Nitrogen limitation media: 3.0 gC/L, 2.79 *10^–4^ gFe/L, nitrogen at concentration listed. Iron limitation media: 3.0 gC/L, 0.5 gN/L, iron at concentration listed.

### Promoter Activity Calculation

Promoter activity was calculated as the change in population GFP per unit time divided by OD leading to the following expression [40,60]:

$$P_{rhlAB}=\frac{1}{OD}\frac{dGFP}{dt}$$

OD and GFP data are smoothed before calculating promoter activity using the 1-D digital filter using the function *filter* in Matlab with a window size of 5 timepoints. This calculation accounts for GFP dilution by cell division.

### Time Lapse of Gene Expression in Swarming Colonies

Swarming motility and GFP expression were monitored at 37°C at 10 min intervals using a custom-made colony visualizer. The images acquired were processed using Matlab to quantify GFP signal and colony density.

### Batch Culture Growth Curves

Starter cultures are inoculated into 3 mL of LB Miller from glycerol stock and incubated overnight at 37°C with shaking. 1 mL of this LB culture is taken, pelleted at 6000 rpm and re-suspended with 1x PBS. Pelleting and re-suspension in PBS is repeated twice before inoculation into the growth media at an OD600 of 0.0025. All growth curves are performed at 37°C in clear flat-bottom BD Falcon 96 well microtiter plates with 150 μL of media per well. Measurements were taken using a Tecan M1000 plate reader (Mannedor, Switzerland) every 10 minutes for the duration of the experiment. Quorum sensing autoinducers HSL and C4HSL used to induce *rhlAB* expression were obtained from Sigma-Aldrich, St. Louis, MO. Unless otherwise indicated, all growth curves are aligned to have OD 0.01 occur at 10 hours. The lag time for each growth curves is determined during this analysis and reported in S2 Table.

### Mathematical Modeling and Parameter Fitting

The mathematical model was implemented as a system of ordinary differential equations in Matlab (the Mathworks, Natick, MA) and solved numerically using the *ode45* function. Parameter fitting was carried out using the *fminbnd* function in a step-wise manner. The OD time series were fitted initially, since these data are independent of the GFP time series data. The goal function to be minimized was defined as:

$$err_{OD}={\sum_{i=1}^{N}\left(\text{log}\left(\frac{X_{i}}{OD_{i}}\right)\right)}^{2}$$

where *i* ∈ {1, *N*} represents all the data points used for the fit. The GFP data was fitted by first calculating the promoter activity from the data as explained in the main text followed by fitting with *fminbnd* to minimize the following function

$$err_{GFP}={\sum_{i=1}^{N}\left(P_{i}-p_{i}\right)}^{2}$$

where *P*_*i*_ is the promoter activity calculated from the data and *p*_*i*_ is the promoter activity predicted by the model. Parameter sensitivity was performed for both the bacterial growth and *rhlAB* expression models and results are reported in S4 and S5 Tables.

### Nutrient Depletion Experiment

Starter cultures and inoculation were performed as in Batch Culture Growth curves. Cells were grown to exponential phase in media with 3.0 gC/L, 0.5 gN/L and 2.79 *10^–4^ gFe/L in 88 wells of a 96 well plate. All wells were harvested and the cells were pelleted and washed with PBS as in Batch culture growth curves. The harvested cells were then split and re-suspended either 1.2 mL of media without nitrogen (3.0 gC/L, 2.79 *10^–4^ gFe/L) or 1.2 mL media without carbon (0.5 gN/L, 2.79 *10^–4^ gFe/L) and grown in a 96 well plate in a Tecan M1000 plate reader as described in Batch Culture Growth curves.

### *P*. *aeruginosa* Swarming Colonies

Swarming assays were performed as previously described [36]. Nitrogen was supplemented with ammonium sulfate at 0.5gN/L. Iron was supplemented using iron(II) sulfate at 2.79*10^–4^ gFe/L. Quorums sensing autoinducers were added at the listed concentrations from liquid stock solutions.

## Supporting Information

S1 Table Number of biological replicates and technical replicates for the representative growth curves shown. (PDF)

S2 Table Lag times for each growth curve. Lag times are reported in hours and are the median lag time for the technical replicates.
(PDF)

S3 Table List of all free parameters in the model of bacterial growth and gene expression. (PDF)

S4 Table Parameter sensitivity of the bacterial growth model. The model was fit using a randomly selected two thirds of the data from the carbon, nitrogen and iron limitation curves shown in Fig 2. This fitting was repeated 100 times and the maximum value and minimum value from this fitting are reported here as well as the fitted variable value when all the data was used to fit.
(PDF)

S5 Table Parameter sensitivity of the *rhlAB* expression model. The model was fit using a randomly selected 4 replicates per growth curve of the carbon, nitrogen and iron limitations (replicates were selected from the representative experiments displayed in the figures) shown in Fig 2. This fitting was repeated 100 times and the maximum value and minimum value from this fitting are reported here as well as the fitted variable value when all the data was used to fit.
(PDF)

S1 Fig Autofluorescence correction. A. Data used to in the linear regression that is used to correct the P_*rhlAB*_ signal. All data is from the WT strain that expresses no fluorescent proteins under different nutrient limitations. Low iron data is from media where iron alone or iron and carbon or iron and nitrogen are limiting throughout growth. Autofluorescence has a similar relationship to OD across all limitations. B. Population level GFP raw data (black) and after correction for autofluorescence (red). See Promoter Activity Calculation for calculation. The correction for autofluorescence in the GFP channel significantly changes the observed GFP.
(EPS)

S2 Fig Single cell gene expression analysis. A. OD of *P*. *aeruginosa* populations in synthetic media using growth curve synchronization method of inoculation [33]. #1–9 are a dilution series of initial inoculum with 1 being the highest initial inoculum (OD 0.01) and then two-fold dilutions were used to create 2–9. Population with a higher initial inoculum are at a more advanced growth stage at the time of harvesting B. Total GFP from P_*rhlAB*_ *-gfp* promoter of populations in A. Populations further along in growth have higher levels of GFP expression C. Histograms of log(GFP/DSRED) ratio for each population in A. DSRED is expressed from a constitutive promoter inserted in the genome and is used as a proxy for cell size. The expression of *rhlAB* is not bimodal, but increases as a single peak as growth progresses. Colors correspond to populations #1–9 in A and B.
(EPS)

S3 Fig Nutrient yields. All data is taken from the growth curves shown in Fig 2A–2C. A-B Note that the line intersects close to 0 OD and 0 initial concentration for both nitrogen and carbon indicating that trace quantities of these nutrients are not present in the media. A. Yield of OD per gC/L in the media. The constant slope indicates a constant yield for carbon. Blue 0.5 gC/L, light blue 0.25 gC/L, orange 0.125 gC/L, red 0.0625 gC/L. B. Yield of OD per gN/L in the media. The constant slope indicates a constant yield for nitrogen. Blue 0.0625 gN/L, light blue 0.0313 gN/L, orange 0.0156 gN/L, red 0.0078 gN/L. C. Yield of OD per gFe/L supplemented to the in the media. The grey point represents the yield for growth on trace iron alone with no iron supplemented to the media. Absolute value of the point where the regression intersects the X-axis is the trace iron concentration. Supplemented iron concentrations: Blue 8.712 x 10^–6^ gFe/L, light blue 4.356 x 10^–6^ gFe/L, orange 2.178 x 10^–6^ gFe/L, red 4.356 x 10^–6^ gFe/L, gray 0.0 gFe/L. For all panels: yield of OD produced is determined from the end of phase I by hand for all points shown and the black line represents a linear regression for the data.
(EPS)

S4 Fig Growth with no iron supplementation. The population was grown in media with 3.0 gC/L and 0.5 gN/L with no iron added to the media. As predicted from the yield calculations in S3C Fig the population is able to grow and sustain exponential growth at the same growth rate as the populations supplemented with iron. The population also reaches the OD predicted by the calculated amount of trace S3C Fig.
(EPS)

S5 FigSchematic of mathematical model of bacterial growth and *rhlAB* promoter activity.Schematic summarizing the growth model where carbon, nitrogen and iron are taken up into the cell and used to produce biomass. Intracellular pools of nitrogen and iron are used for growth when these nutrients are depleted from the extracellular environment. The three nutrients in the model (C, N, Fe) each have their own yield, which determines how much biomass (X) can be produced per gram of each nutrient. The intracellular pools of nitrogen and iron also have yields to determine what fraction of the intracellular pool is needed to produce a unit of biomass (X). The three components of the *rhlAB* promoter activity model are also summarized here. Density dependent regulation is scaled by q_D_, induction by limited growth (calculated from the growth model) is scaled by q_R_, and shut down of promoter activity when growth becomes extremely limited is determined by k_g_ and the hill coefficient h. Note that the for simplicity the displayed *rhlAB* promoter activity components have been simplified such that q_R_ and ${\left(k_{g}\times \frac{\mu_{\text{max}}}{\mu }\right)}^{h}$ represent $q_{R}\left(\frac{\mu_{\text{max}}\left('\right)}{\mu }-1\right)$ and $\frac{1}{1+{\left(k_{g}\times \frac{\mu_{\text{max}}}{\mu }\right)}^{h}}$ respectively. Together the *rhlAB* promoter activity components result in density dependent promoter activity during phase I, limited growth induced promoter activity during phase II, and an eventual shut down of promoter activity during prolonged phase II growth. Also note that C, N and Fe can all be experimentally manipulated and that X and *rhlAB* activity are measured experimentally using OD and GFP respectively. State variables, C, N, Fe, N_i_, Fe_i_, and X, are shown in bold. Components not explicitly modeled, C_i_ (intracellular carbon) and RL (rhamnolipids), are shown in gray.(EPS)

S6 Fig Effect of quorum sensing concentration on *rhlAB* expression. A. Basal *rhlAB* expression by GFP from the P_*rhlAB*_-gfp construct with varying levels of C4HSL and HSL autoinducers in synthetic media. Increasing levels of autoinducers result in increased levels of basal promoter activity. Higher levels of one signal do not directly compensate for low levels of the other. B. Maximual *rhlAB* expression by GFP from the P_*rhlAB*_-gfp construct with varying levels of C4HSL and HSL autoinducers. Maximal level of expression also increases with increasing levels of autoinducers and again there is not a direct compensation of one for the other. All Cultures were grown in Synthetic media with 3.0 gC/L, 0.5 gN/L and no iron supplementation.
(EPS)

S7 Fig Quorum sensing scales *rhlAB* promoter activity during iron and nitrogen starvation. A. Growth of populations in iron limitation media (0 gFe/L supplemented iron) at different concentrations of quorum sensing signals 1 X = 1 μM C12HSL and 5 μM C4HSL. Growth is similar in different quorum sensing concentrations B. GFP production in iron limiting media in different concentrations of quorum sensing signals. Higher levels of signal result in higher levels of expression. C. Promoter activity in iron limitation media in different concentrations of quorum sensing signals. Promoter activity during balanced growth (~0-6h) and promoter activity during starvation (~6-30h) are both scaled by the level of quorum sensing signals. D. Growth of populations in nitrogen limitation media (0.03 gN/L) at different concentrations of quorum sensing signals 1 X = 1 μM C12HSL and 5 μM C4HSL. Growth is similar in different quorum sensing concentrations E. GFP production in nitrogen limiting media in different concentrations of quorum sensing signals. Higher levels of signal result in higher levels of GFP expression. F. Promoter activity in nitrogen limitation media in different concentrations of quorum sensing signals. Promoter activity during balanced growth (~0-10h) and promoter activity during starvation (~10-30h) are both scaled by the level of quorum sensing signals.
(EPS)

S8 Fig Effects of Quorum sensing signals and nutrient concentrations on swarming colony formation. A. WT *P*. *aeruginosa* swarming colony morphology in standard media. B. Branching morphology is affected when additional nitrogen is added to the media (0.5 gN/L by ammonium sulfate). This could be due to decreased overall rhamnolipid production C. Swarming cooperation is prevented by the addition of iron to the media (2.79*10^–4^ gFe/L by iron(II) sulfate). Lack of iron limitation reduces overall rhamnolipid production preventing the colony from swarming. D-F The coverage of WT swarming colonies is reduced with increasing concentrations of quorum sensing signals added to the media (QS 1x = 1 μM C12HSL and 5 μM C4HSL). This could be due to overproduction of rhmanolipids or over production of other quorum sensing regulated secreted products such as exopolysaccharides.
(TIFF)
