# Supplementary material for: Integration of Metabolic and Quorum Sensing Signals Governing the Decision to Cooperate in a Bacterial Social Trait
Source: PLoS Comput Biol. 2015 Jun 23;11(6):e1004279. doi: 10.1371/journal.pcbi.1004279 (PMC4477906; doi:10.1371/journal.pcbi.1004279)
Supplement: S1 Table — (PDF) [file pcbi.1004279.s001.pdf]

Table S1

Experiment	Carbon Limitation	Nitrogen Limitation	Iron Limitation	Nutrient Depletion	Quorum Sensing
Replicate Wells for displayed experiment	6	12	6	6	6
Biological Replicates	3	3	3	4	3
