# Supplementary material for: Integration of Metabolic and Quorum Sensing Signals Governing the Decision to Cooperate in a Bacterial Social Trait
Source: PLoS Comput Biol. 2015 Jun 23;11(6):e1004279. doi: 10.1371/journal.pcbi.1004279 (PMC4477906; doi:10.1371/journal.pcbi.1004279)
Supplement: S2 Table — Lag times are reported in hours and are the median lag time for the technical replicates. (PDF) [file pcbi.1004279.s002.pdf]

Table S2

Growth Curve	Lag Phase [hours]
**Carbon Limitation**	
0.50 gC/L	4.4228
0.25 gC/L	3.9737
0.125 gC/L	3.6973
0.063 gC/L	3.0001
**Nitrogen Limitation**	
0.0625 gN/L	3.1000
0.0312 gN/L	4.3812
0.0156 gN/L	2.6570
0.0078 gN/L	3.2814
**Iron Limitation (Supplemented Concentration)**	
$6.981 * 10^{-5}$ gFe/L	2.7400
$3.490 * 10^{-5}$ gFe/L	2.6322
$1.745 * 10^{-5}$ gFe/L	2.6269
$8.726 * 10^{-6}$ gFe/L	3.2804
$4.362 * 10^{-6}$ gFe/L	3.1083
$2.181 * 10^{-6}$ gFe/L	3.5053
$1.089 * 10^{-6}$ gFe/L	4.5329
0 gFe/L	4.6104
**Quorum Sensing**	
4 X	6.5532
2 X	6.4149
1 X	6.4797
½ X	6.2006
¼ X	5.4931
0	4.1117
