# Supplementary material for: Integration of Metabolic and Quorum Sensing Signals Governing the Decision to Cooperate in a Bacterial Social Trait
Source: PLoS Comput Biol. 2015 Jun 23;11(6):e1004279. doi: 10.1371/journal.pcbi.1004279 (PMC4477906; doi:10.1371/journal.pcbi.1004279)
Supplement: S3 Table — (PDF) [file pcbi.1004279.s003.pdf]

Table S3

Parameter	Units	Represents	Value in displayed fit
μ_{max}	h^{-1}	Maximum growth rate when N>0	0.3341
Y_C	OD/gC	Yield for carbon	0.6516
k_d	h^{-1}	decay when C=0	0.0062
Y_N	OD/gN	Yield for nitrogen	4.1627
μ_{max}'	h^{-1}	Maximum growth rate when N = 0	0.0616
Y_{Ni}	OD/N_i	Yield for internal nitrogen	2.0011
Y_{Fe}	OD/gFe	Yield for iron	$3.8952 * 10^3$
Y_{Fei}	OD/I_i	Yield for internal iron	4.9892
q_D	GFP/OD	Density dependent scaling factor	$1.8542 * 10^4$
q_{RN}	GFP	Nitrogen starvation upregulation scaling factor	$7.4541 * 10^3$
k_{gN}	-	Fraction of μ_{max} in nitrogen starvation when down regulation begins	0.1667
h_N	-	Hill coefficient nitrogen starvation down regulation	5.8725
q_{RFe}	GFP	Iron starvation upregulation scaling factor	$8.5933 * 10^3$
k_{gFe}	-	Fraction of μ_{max} in iron starvation when down regulation begins	0.3293
h_{Fe}	-	Hill coefficient iron starvation down regulation	4.9892
