# Supplementary material for: Integration of Metabolic and Quorum Sensing Signals Governing the Decision to Cooperate in a Bacterial Social Trait
Source: PLoS Comput Biol. 2015 Jun 23;11(6):e1004279. doi: 10.1371/journal.pcbi.1004279 (PMC4477906; doi:10.1371/journal.pcbi.1004279)
Supplement: S4 Table — The model was fit using a randomly selected two thirds of the data from the carbon, nitrogen and iron limitation curves shown in Fig 2. This fitting was repeated 100 times and the maximum value and minimum value from this fitting are reported here as well as the fitted variable value when all the data was used to fit. (PDF) [file pcbi.1004279.s004.pdf]

Table S4

Variable	μ_{max}	Y_C	k_d	Y_N	μ_{max}'	Y_{Ni}	Y_{Fe}	Y_{Fei}
Units	h^{-1}	OD/gC	h^{-1}	OD/gN	h^{-1}	OD/N_i	OD/gFe	OD/I_i
**Fit with all data**	**0.3341**	**0.6516**	**0.0062**	**4.1627**	**0.0616**	**2.0011**	**$3.8952 * 10^3$**	**4.9892**
Highest parameter value fitted with 2/3 data	0.3464	0.6611	0.0061	4.2644	0.0614	2.0492	$3.6548 * 10^3$	5.1533
Lowest parameter value fitted with with 2/3 data	0.3243	0.6387	0.0059	4.1799	0.0601	1.9234	$3.9305 * 10^3$	5.2690
