# Supplementary material for: Integration of Metabolic and Quorum Sensing Signals Governing the Decision to Cooperate in a Bacterial Social Trait
Source: PLoS Comput Biol. 2015 Jun 23;11(6):e1004279. doi: 10.1371/journal.pcbi.1004279 (PMC4477906; doi:10.1371/journal.pcbi.1004279)
Supplement: S5 Table — The model was fit using a randomly selected 4 replicates per growth curve of the carbon, nitrogen and iron limitations (replicates were selected from the representative experiments displayed in the figures) shown in Fig 2. This fitting was repeated 100 times and the maximum value and minimum value from this fitting are reported here as well as the fitted variable value when all the data was used to fit. (PDF) [file pcbi.1004279.s005.pdf]

Table S5

Variable	q_D	q_{RN}	k_{gN}	h_N	q_{RFe}	k_{gFe}	h_{Fe}
Units	GFP/OD	GFP	-	-	GFP	-	-
**Fit with all data**	**$1.8542 * 10^4$**	**7.4541** $* 10^3$	**0.1667**	**5.8725**	**$8.5933 * 10^3$**	**0.3293**	**1.9158**
Highest parameter value fitted with 4 replicates each	$1.9005 * 10^4$	$9.4672 * 10^3$	0.1765	5.7399	$8.2950 * 10^3$	0.3193	1.9554
Lowest parameter fitted with with 4 replicates each	$1.7595 * 10^4$	$6.2586 * 10^3$	0.1662	5.2734	$7.1706 * 10^3$	0.2926	1.9274
