# Supplementary figures and images for: Integration of Metabolic and Quorum Sensing Signals Governing the Decision to Cooperate in a Bacterial Social Trait
Source: PLoS Comput Biol. 2015 Jun 23;11(6):e1004279. doi: 10.1371/journal.pcbi.1004279 (PMC4477906; doi:10.1371/journal.pcbi.1004279)

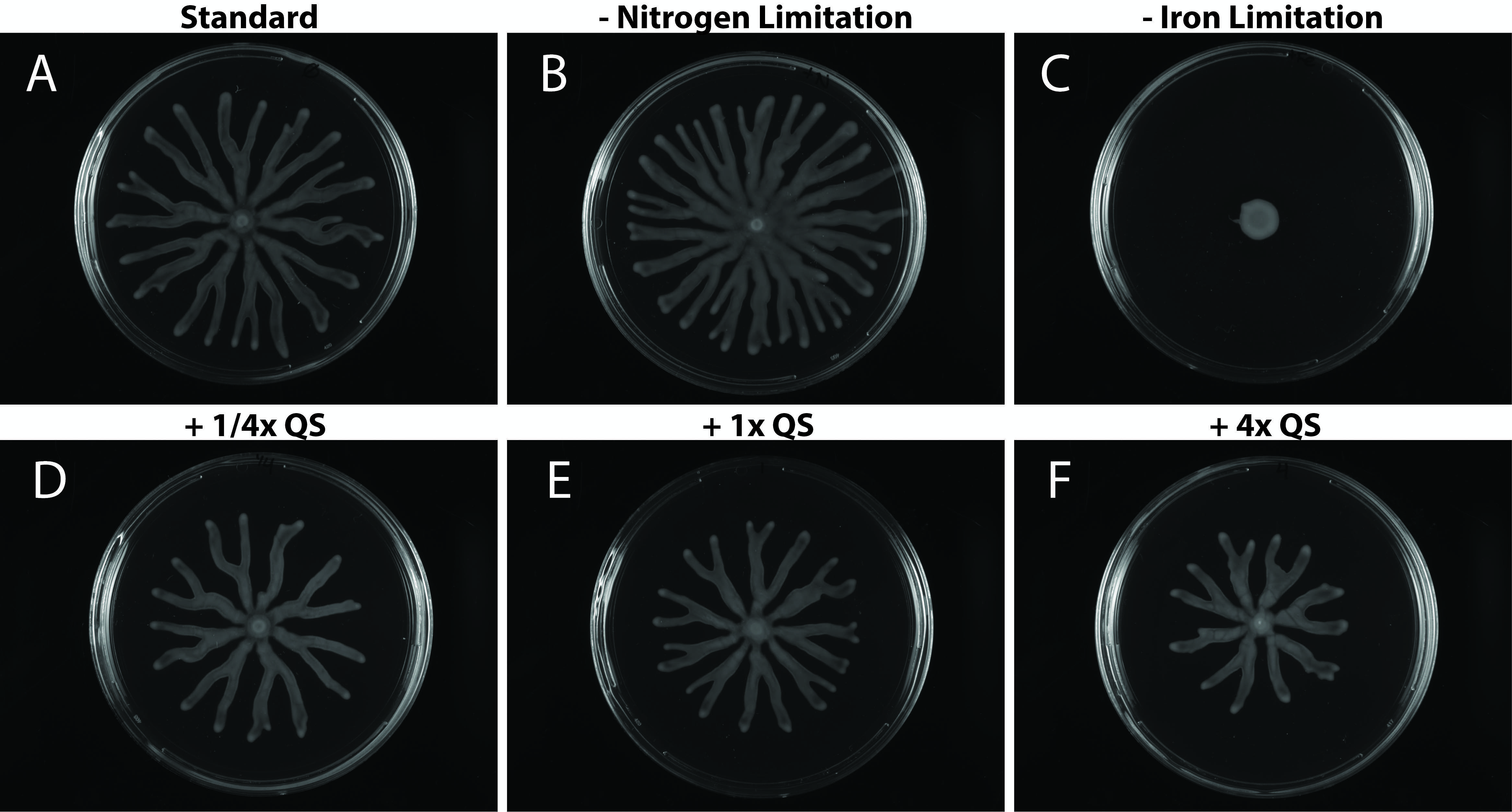

Supplement: S8 Fig — A. WT P. aeruginosa swarming colony morphology in standard media. B. Branching morphology is affected when additional nitrogen is added to the media (0.5 gN/L by ammonium sulfate). This could be due to decreased overall rhamnolipid production C. Swarming cooperation is prevented by the addition of iron to the media (2.79*10–4 gFe/L by iron(II) sulfate). Lack of iron limitation reduces overall rhamnolipid production preventing the colony from swarming. D-F The coverage of WT swarming colonies is reduced with increasing concentrations of quorum sensing signals added to the media (QS 1x = 1 μM C12HSL and 5 μM C4HSL). This could be due to overproduction of rhmanolipids or over production of other quorum sensing regulated secreted products such as exopolysaccharides. (TIFF) [file pcbi.1004279.s013.tiff]
